# All‐Solid‐State Planar Sodium‐Ion Microcapacitors with Multidirectional Fast Ion Diffusion Pathways

**DOI:** 10.1002/advs.201902147

**Published:** 2019-10-04

**Authors:** Shuanghao Zheng, Sen Wang, Yanfeng Dong, Feng Zhou, Jieqiong Qin, Xiao Wang, Feng Su, Chenglin Sun, Zhong‐Shuai Wu, Hui‐Ming Cheng, Xinhe Bao

**Affiliations:** ^1^ Dalian National Laboratory for Clean Energy Dalian Institute of Chemical Physics Chinese Academy of Sciences 457 Zhongshan Road Dalian 116023 China; ^2^ State Key Laboratory of Catalysis Dalian Institute of Chemical Physics Chinese Academy of Sciences 457 Zhongshan Road Dalian 116023 China; ^3^ University of Chinese Academy of Sciences 19A Yuquan Road, Shijingshan District Beijing 100049 China; ^4^ Shenyang National Laboratory for Materials Science Institute of Metal Research Chinese Academy of Sciences 72 Wenhua Road Shenyang 110016 China; ^5^ Shenzhen Geim Graphene Center Tsinghua–Berkeley Shenzhen Institute 1001 Xueyuan Road Shenzhen 518055 China

**Keywords:** energy storage, flexible, in‐plane geometry, ionogel electrolytes, sodium‐ion microcapacitors

## Abstract

With the relentless development of smart and miniaturized electronics, the worldwide thirst for microscale electrochemical energy storage devices with form factors is launching a new era of competition. Herein, the first prototype planar sodium‐ion microcapacitors (NIMCs) are constructed based on the interdigital microelectrodes of urchin‐like sodium titanate as faradaic anode and nanoporous activated graphene as non‐faradaic cathode along with high‐voltage ionogel electrolyte on a single flexible substrate. By effectively coupling with battery‐type anode and capacitor‐type cathode, the resultant all‐solid‐state NIMCs working at 3.5 V exhibit a high volumetric energy density of 37.1 mWh cm^−3^ and an ultralow self‐discharge rate of 44 h from *V*_max_ to 0.6 *V*_max_, both of which surpass most reported hybrid micro‐supercapacitors. Through tuning graphene layer covered on the top surface of interdigital microelectrodes, the NIMCs unveil remarkably enhanced power density, owing to the establishment of favorable multidirectional fast ion diffusion pathways that significantly reduce the charge transfer resistance. Meanwhile, the as‐fabricated NIMCs present excellent mechanical flexibility without capacitance fade under repeated deformation, and electrochemical stability at a high temperature of 80 °C because of using nonflammable ionogel electrolyte and in‐plane geometry. Therefore, these flexible planar NIMCs with multidirectional ion diffusion pathways hold tremendous potential for microelectronics.

## Graphical abstract

All‐solid‐state planar sodium‐ion microcapacitors are constructed on a single substrate based on faradaic sodium titanate as anode and non‐faradaic activated graphene as cathode along with nonflammable high‐voltage ionogel electrolyte. The resultant microcapacitors possess the merit of multidirectional fast ion diffusion pathways and show high volumetric performance, remarkable flexibility, and high‐temperature stability.

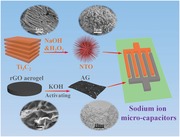

Microscale electrochemical energy storage devices have been extensively acknowledged as key power sources for miniaturized smart and integrated electronics, such as remote sensors, microrobots, and self‐powered microsystems.^1^, ^2^, ^3^, ^4^, ^5^, ^6^, ^7^, ^8^ In principle, microscale electrochemical energy storage devices are primarily referred to microbatteries (MBs) and micro‐supercapacitors (MSCs), both of which, in particular, with an in‐plane geometry, can be directly coupled with integrated circuits as standalone or complement power sources.^9^, ^10^, ^11^, ^12^ Among them, lithium‐ion MBs (LIMBs) can inherently deliver a high energy density of 200 mWh cm^−3^ or more,^13^, ^14^, ^15^, ^16^, ^17^ but have many restrictions such as limited cycling lifespan, poor safety, and low power density below 10 mW cm^−3^ induced by the slow lithium‐ion diffusion kinetics.^18^, ^19^, ^20^ In contrast, MSCs can fully reversibly adsorb/desorb electrolyte ions or perform fast faradaic reaction at the interface of electrode and electrolyte, affording high power density of up to 1000 W cm^−3^, fast charge and discharge rate, and longevity,^21^, ^22^, ^23^ while they suffer from low energy density, usually less than 5 mWh cm^−3^.^24^, ^25^, ^26^ As a consequence, neither LIMBs nor MSCs can simultaneously meet the extreme requirements of miniaturized electronics for high energy and power densities.

To solve the above issue, one reliable strategy is to develop hybrid ion microcapacitors (HIMCs), consisting of a battery‐type electrode and a supercapacitor‐type electrode, which can synchronously combine the merits of high energy density of MBs and high power density of MSCs, and effectively bridge the huge gap between them.^27^, ^28^, ^29^, ^30^ Therefore, HIMCs are regarded as a highly competitive class of next‐generation microscale energy storage devices, e.g., lithium‐ion microcapacitors (LIMCs).^11^, ^31^, ^32^, ^33^ Typically, the reported planar LIMCs constructed with insertion‐type lithium titanate and adsorption‐type porous graphene exhibited high volumetric energy density and outstanding flexibility,^11^ showing great potential for flexible microelectronic devices, but their massive applications would be likely prevented by the restrained source and rising cost of lithium.^30^, ^34^ Alternatively, much attentions have been devoted to sodium‐ion energy storage devices, thanks to the abundant sodium resource, low cost, and comparable electrochemical properties of sodium to lithium.^27^, ^31^, ^35^, ^36^ However, sodium‐ion microcapacitors (NIMCs) with in‐plane geometry have not yet been reported so far due to the lack of cost‐effective fabrication method and reasonable design of microelectrodes.

Herein, we, for the first time, reported the assembly of all‐solid‐state flexible planar NIMCs with an urchin‐like sodium titanate (NTO) anode and nanoporous activated graphene (AG) cathode (denoted as NTO//AG‐NIMCs). The interdigital microelectrodes have a sandwich‐like structure, outstanding flexibility, and excellent electronic conductivity (200 S cm^−1^ for anode and 100 S cm^−1^ for cathode). By designing a cover layer on the patterned microelectrodes, it was unveiled that NTO//AG‐NIMCs exhibited multidirectional ion diffusion mechanism that the flow of ions can rapidly pass through two side surfaces and the top surface of the microelectrodes to boost high‐power capability. The as‐fabricated NTO//AG‐NIMCs operated well in a high‐voltage ionogel electrolyte with 3.5 V, and showed an ultralow self‐discharge rate of up to 44 h from *V*_max_ to 0.6 *V*_max_, a high energy density of 37.1 mWh cm^−3^ and a superior power density of 1.2 W cm^−3^. Meanwhile, these microdevices exhibited outstanding flexibility and high‐temperature resistance of up to 80 °C.

The fabrication of NTO//AG‐NIMCs was systemically presented in **Figure** **1**, along with the preparation and characterization of negative electrode NTO and positive electrode AG. First, urchin‐like NTO was selected as anode material because of low potential (<0.5 V vs Na/Na^+^) for sodium insertion/extraction, high capacity, and stable structure during charge and discharge processes.^37^, ^38^ As shown in Figure 1a, the urchin‐like NTO was prepared from the simultaneous oxidation and alkalization of Ti_3_C_2_ MXene, obtained from the HF etching of layered Ti_3_AlC_2_.^39^ The characteristic peak of NTO at 9.8° was upshifted (Figure S1, Supporting Information), showing an extended interlayer spacing of 0.9 nm. Transmission electron microscope (TEM) images (Figure S2, Supporting Information) exhibit the porous structure of twining and elongated NTO nanoribbons. Such features of NTO are beneficial for rapid Na^+^ incorporation/deintercalation. Second, nanoporous AG as cathode was synthesized from the KOH activation of 3D reduced graphene oxide (rGO) aerogels fabricated by self‐propagating combustion (Figure 1b; Figures S3 and S4, Supporting Information). Scanning electron microscope (SEM) and TEM images (Figure 1b, downward side) revealed the morphological transformation from 3D macroporous graphene oxide (GO) and rGO aerogels to mesoporous AG. Importantly, AG showed a large surface area of 2380 m^2^ g^−1^ and mesoporous structure with a typical average size of 2 nm (Figure S5, Supporting Information), which are favorable for enlarging the accessibly available active surface for charge storage, and facilitating the diffusion of electrolyte ions. Third, NTO//AG‐NIMCs based on interdigitated microelectrode patterns of NTO and AG (Figure 1c) were constructed on a flexible substrate using a mask‐assisted filtration strategy. To form highly conductive skeleton, high‐conducting electrochemically exfoliated graphene (EG, 1000 S cm^−1^; Figure S6, Supporting Information) was chosen as 2D flexible support, conducting additive, and metal‐free current collectors for the fabrication of asymmetric microelectrodes. The asymmetric interdigitated microelectrodes were fabricated by sequential layer‐by‐layer deposition of EG as the bottom layer, electrode materials as the middle layer, and EG as the ultrathin and half‐baked top layer, to form a highly stable sandwich‐like electrode structure (see details in Experimental Section). Cross‐sectional SEM images (Figure 1c, right side) of the NTO anode and AG cathode displayed typical thicknesses of ≈5 and ≈10 µm, respectively. Moreover, the interdigital microelectrodes exhibited hierarchically sandwich‐like compact configurations, e.g., EG/NTO–EG/EG and EG/AG–EG/EG, where the conductive crosslinking frameworks of EG nanosheets are helpful for the formation of the NTO or AG microelectrodes, and have strong adhesion to the substrate. As demonstrated in Figure 1d–h, when the interdigital microelectrodes were subjected to severe mechanical bending of the coiling and uncoiling processes for several times, they did not fracture and had no delamination from the substrate (Figure S7, Supporting Information), indicative of excellent structural integrity. Furthermore, the as‐fabricated anode and cathode patterns showed outstanding electronic conductivities of ≈200 and ≈100 S cm^−1^ (Figure S8, Supporting Information), respectively. After the anode and cathode microelectrodes were bent for 1000 cycles, the electrical conductivities with a slight fluctuation were almost unchanged (Figure S9, Supporting Information), indicating the stable structure of the microelectrodes.

**Figure 1:**
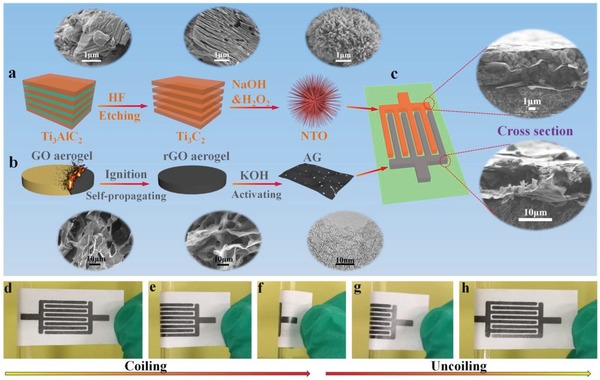
Scheme of the fabrication of NTO//AG‐NIMCs along with the preparation and characterization of NTO and AG. a) The schematic preparation of urchin‐like NTO from the alkalization and oxidation of Ti_3_C_2_ MXene, obtained from etched Ti_3_AlC_2_. Insets at the upward side are the corresponding SEM images of Ti_3_AlC_2_, Ti_3_C_2_ MXene, and NTO. b) AG synthesized from the activation of rGO aerogels, derived from the self‐propagation combustion of GO aerogels. Insets at the downward side are SEM images of GO and rGO aerogels, and TEM image of AG. c) Schematic illustration of NTO//AG‐NIMCs based on interdigital patterns of NTO and AG microelectrodes. Insets are the cross‐sectional SEM images of anode and cathode. d–h) Optical images showing the d–f) coiling and g–h) uncoiling processes of NTO//AG‐NIMCs.

To address the serious problems of conventional organic electrolytes with flammability and low safety,^40^, ^41^ we developed a new nonflammable ionogel electrolyte based on bis(trifluoromethanesulfonyl) imide sodium salt (NaTFSI), 1‐butyl‐1‐methyl‐pyrrolidinium bis(trifluoromethanesulfonyl) imide (P_14_TFSI), and poly(vinylidene difluoride‐*co*‐hexafluoropropylene) (PVDF‐HFP) (NaTFSI–P_14_TFSI–PVDF‐HFP) (**Figure** **2**a). The oxidation stability of the ionogel electrolyte was evaluated via linear sweep voltammetry (LSV) at 2 mV s^−1^. As shown in Figure 2b, a very low oxidation current was observed until the potential reached to 5.0 V, indicative of a large stable voltage window of this ionogel electrolyte. Importantly, the ionogel electrolyte has another advantageous nature of nonflammability, which cannot be burnt in flammable test even ignited for 30 s (Figure 2c,d), suggestive of superior thermal stability. However, commercial sodium‐ion gel electrolyte, NaPF_6_ in diglyme mixed with PVDF‐HFP, was easily burnt once it was ignited (Figure S10, Supporting Information), further showing the robust safety of the as‐prepared ionogel electrolyte. After the ionogel electrolyte was coated on the surface of microelectrodes and solidified, all‐solid‐state NTO//AG‐NIMCs were obtained (Figure 2a).

**Figure 2:**
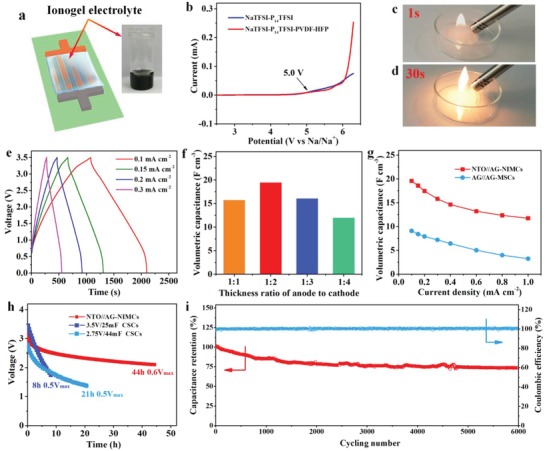
The property of ionogel electrolyte and electrochemical performance of NTO//AG‐NIMCs. a) Schematic of ionogel‐based NTO//AG‐NIMCs. Inset is the photograph of NaTFSI–P_14_TFSI–PVDF‐HFP ionogel electrolyte. b) LSV curves of NaTFSI–P_14_TFSI and NaTFSI–P_14_TFSI–PVDF‐HFP ionogel electrolyte at 2 mV s^−1^. c,d) The nonflammable test of ionogel electrolyte. e) GCD profiles of NTO//AG‐NIMCs tested from 0.1 to 0.3 mA cm^−2^. f) Volumetric capacitance with different thickness ratios of anode to cathode. g) Volumetric capacitance of NTO//AG‐NIMCs and AG//AG‐MSCs from 0.1 to 1 mA cm^−2^. h) Self‐discharge curves of NTO//AG‐NIMCs in comparison with commercially available supercapacitors. i) Long‐term cycling performance of NTO//AG‐NIMCs for 6000 times obtained at 0.8 mA cm^−2^.

The electrochemical performance of NTO//AG‐NIMCs was examined by galvanometric charge and discharge (GCD) and cyclic voltammetry (CV) measurements at a voltage of 3.5 V. The gradual line of GCD profiles between 0.5 and 3 V resulted from Na^+^ ion insertion/extraction at NTO anode and TFSI^−^ ion adsorption/desorption at AG cathode during the charge and discharge process (Figure 2e), which was in good line with the wide redox peaks of CV curves (Figure S11, Supporting Information). It was implied that the all‐solid‐state NTO//AG‐NIMCs were successfully constructed with suitable match of faradaic NTO and non‐faradaic AG. It was noted from Figure S12 (Supporting Information) that the contribution of the EG current collectors to the total capacitance of NTO//AG‐NIMCs was almost negligible (0.6%). Furthermore, it is disclosed that NTO//AG‐NIMCs, with an optimal thickness ratio of 1:2 (Figure 2f), offered the highest volumetric capacitance of 19.5 F cm^−3^, which was much higher than those microdevices with other ratios, e.g., 15.8 F cm^−3^ for 1:1, 16.1 F cm^−3^ for 1:3, 12 F cm^−3^ for 1:4, and superior to AG//AG‐MSCs (9.1 F cm^−3^) at the same current density of 0.1 mA cm^−2^ (Figure 2g; Figures S13 and S14, Supporting Information). Moreover, at the high current density of 1 mA cm^−2^, the volumetric capacitance of NTO//AG‐NIMCs slightly decreased to 11.7 F cm^−3^ that was equal to 60% of the initial capacitance, apparently outperforming AG//AG‐MSCs (36%) at the same condition (Figure S14, Supporting Information). This result is attributed to the synergistic combination of highly effective conductive framework in microelectrodes, high‐performance active materials of NTO and AG, and high‐voltage ionogel electrolyte with a remarkable ionic conductivity of 0.5 mS cm^−1^.

For practical application, MSCs, especially operating at a high voltage, urgently demand ultralow self‐discharge rate, which was largely ignored in most studies. To highlight this feature, we precharged NTO//AG‐NIMCs to *V*_max_ = 3.5 V and recorded their self‐discharge profiles, as shown in Figure 2h. It is revealed that the self‐discharge time of NTO//AG‐NIMCs required from *V*_max_ to 0.6 *V*_max_ was as long as 44 h, much longer than those of commercial supercapacitors (CSCs), for instance, 3.5 V/25 mF CSCs (8 h, 0.5 *V*_max_) and 2.75 V/44 mF CSCs (21 h, 0.5 *V*_max_).^24^ In addition, NTO//AG‐NIMCs, carried out by GCD at 0.8 mA cm^−2^, showed long‐term cycling stability with an acceptable capacitance retention of 75% after 6000 cycles (Figure 2i).

It is reported that the in‐plane geometry of the interdigitated devices is different from the sandwich‐like stacked geometry in conventional supercapacitors. The interdigital in‐plane MSCs are free of separators, in which two side surfaces and the upper surface of microelectrodes are completely immersed in the electrolyte (Figure 2a). To demonstrate the importance of in‐plane geometry, we constructed NTO//AG‐NIMCs based on the microelectrodes with a tight EG cover (denoted as NTO//AG‐NIMCs‐C), and compared the electrochemical performance with NTO//AG‐NIMCs without EG cover. SEM images of NTO//AG‐NIMCs display the existence of substantial observable microchannels at the upper surface in the NTO and AG microelectrodes (**Figure** **3**a,b), while in the NTO//AG‐NIMCs‐C, the microelectrodes were fully overlapped by the uniform EG layer (Figure 3c,d). As a result, the NTO//AG‐NIMCs at 0.2 mA cm^−2^ delivered a higher volumetric capacitance of 17.4 F cm^−3^ than the NTO//AG‐NIMCs‐C (Figure 3e, 13.7 F cm^−3^). This is because the electrolyte ions cannot efficiently diffuse into the deep interior of microelectrodes due to the restriction of the upper surface with a tight EG cover. Moreover, NTO//AG‐NIMCs exhibited better rate capability than NTO//AG‐NIMCs‐C. For instance, the capacitance of the NTO//AG‐NIMCs at a high current density of 1.5 mA cm^−2^ showed ≈58% (10.1 F cm^−3^) of the initial capacitance (Figure 3f), overwhelmingly higher than that of the NTO//AG‐NIMCs‐C (33%, 4.5 F cm^−3^). As explained by electrochemical impedance spectroscopy (EIS), NTO//AG‐NIMCs displayed a lower charge transfer resistance of 173 Ω (Figure 3g), while that of NTO//AG‐NIMCs‐C is up to 368 Ω, suggesting that the presence of a tight EG top layer on microelectrodes would partly inhibit the smooth flow of electrolyte ions through the vertical direction (Figure 3h,i). Consequently, it is confirmed that NTO//AG‐NIMCs with in‐plane geometry possess an unique advantage of multidirectional fast ion diffusion pathways from two side surfaces and extra upper surface (Figure 3h,i), in which electrolyte ions can fluently transfer not only in the parallel manner from one side to the other side of microelectrodes, but also in the vertical direction through the upper surface of microelectrodes. In sharp contrast, electrolyte ions in sandwiched supercapacitors with stacked geometry can only flow from one side to the other side through a separator (Figure 3j).

**Figure 3:**
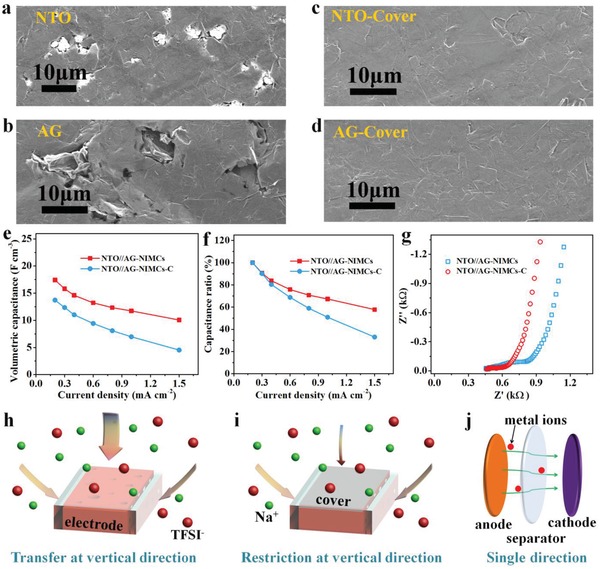
Multidirectional diffusion mechanism of electrolyte ions in NTO//AG‐NIMCs. a–d) Top‐view SEM images of anode and cathode a,b) without and c,d) with EG cover. e) Volumetric capacitance and f) capacitance ratio of NTO//AG‐NIMCs and NTO//AG‐NIMCs‐C as a function of current density. g) EIS spectra of NTO//AG‐NIMCs and NTO//AG‐NIMCs‐C. h) Schematic illustration of the multidirectional ion diffusion pathways at the planar microelectrodes without the EG cover, and i) diagram showing the restriction of ion transfer at the vertical direction due to the presence of the top EG cover. j) Ion transfer with single direction at conventional supercapacitors with a sandwich configuration.

To meet ever‐increasing demands of flexible energy storage devices for smart electronics, the mechanical flexibility of NTO//AG‐NIMCs at different bending conditions was evaluated through GCD profiles (**Figure** **4**a). Even when the microdevice was bent from flat to 180° and even circle states (Figure 4b), the GCD profiles were almost fully overlapped, with a capacitance retention of 100%, indicative of remarkable flexibility of NTO//AG‐NIMCs. After repeatedly bending to 180° for 2000 times, NTO//AG‐NIMCs showed almost unchanged capacitance with a high Coulombic efficiency of 99% (Figure 4c), further demonstrative of the outstanding mechanical stability of NTO//AG‐NIMCs. Moreover, NTO//AG‐NIMCs were easily integrated in series or parallel strategies to satisfy the respective requirements of tailored current and voltage output for smart electronics. A serially connected NTO//AG‐NIMC pack was realized by overlapping NTO and AG microelectrodes of two adjacent NTO//AG‐NIMCs (Figure 4d). The parallelly connected NTO//AG‐NIMCs were integrated via the same NTO and AG microelectrodes (Figure 4e). As a result, from CV curves (Figure 4f) and GCD profiles (Figure 4g), the serially connected NTO//AG‐NIMCs enlarged the voltage window from 3.5 V for one microdevice to 7.0 V for two microdevices, and the parallelly connected NTO//AG‐NIMCs increased the current output and discharge time in proportion, demonstrative of exceptionally robust performance uniformity. Notably, after being charged at a current density of 0.6 mA cm^−2^ for 120 s, single NTO//AG‐NIMC could easily light the display of a “DICP” (Dalian Institute of Chemical Physics) logo for a significant long time of 40 min (Figure 4h), due to its large capacitance, high voltage, and ultralow self‐discharge rate, suggestive of great potential of NTO//AG‐NIMCs for actual applications.

**Figure 4:**
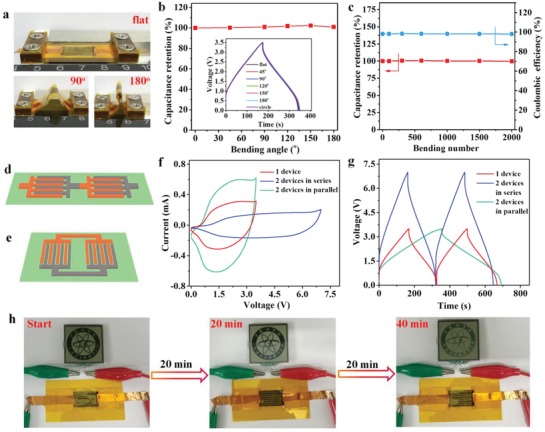
Flexibility and integration of NTO//AG‐NIMCs. a) Photographs of NTO//AG‐NIMCs with flat state and bending states of 90° and 180°. b) Capacitance retention versus bending angle from flat to 180°. Inset is GCD profiles tested at various bending states. c) Capacitance retention versus the bending number. d,e) Schematic diagrams of integrated NTO//AG‐NIMCs in d) series and e) parallel. f) CV curves and g) GCD curves of two NTO//AG‐NIMCs in series and in parallel. h) Optical images of a “DICP” logo powered by one NTO//AG‐NIMC for 40 min after charging for 120 s.

To demonstrate the high safety, we further investigated the electrochemical performance of NTO//AG‐NIMCs at a high temperature of 80 °C (denoted as NTO//AG‐NIMCs‐80), using ionogel electrolyte of NaTFSI–P_14_TFSI–PVDF‐HFP with a thermal stability up to 370 °C (Figure S15, Supporting Information). As shown in **Figure** **5**a, NTO//AG‐NIMCs‐80 showed the similar shapes of GCD profiles to those of the microdevices tested at room temperature (denoted as NTO//AG‐NIMCs‐25), and longer discharge time than NTO//AG‐NIMCs‐25 at the same current density. As expected, NTO//AG‐NIMCs‐80 at 0.3 mA cm^−2^ exhibited enhanced areal and volumetric capacitances of 14.6 mF cm^−2^ and 19.5 F cm^−3^ (Figure 5b), respectively, outperforming those of NTO//AG‐NIMCs‐25 (11.8 mF cm^−2^, 15.8 F cm^−3^) due to the improved ionic conductivity of ionogel electrolyte from 0.5 to 2.8 mS cm^−1^ with the temperature being increased from 25 to 80 °C (Figure S16, Supporting Information). Furthermore, NTO//AG‐NIMCs‐80 revealed that exceptional rate capability with 61% of original capacitance remained at 2 mA cm^−2^. This could be explained by the dramatical decrease of equivalent serial resistance (ESR) from 450 Ω for NTO//AG‐NIMCs‐25 to 126 Ω for NTO//AG‐NIMCs‐80 (Figure 3f; Figure S17, Supporting Information). Remarkably, NTO//AG‐NIMCs‐80 also disclosed a considerable capacitance retention of 75% and a high Coulombic efficiency of >95% after 1500 cycles at 1.5 mA cm^−2^ (Figure 5c).

**Figure 5:**
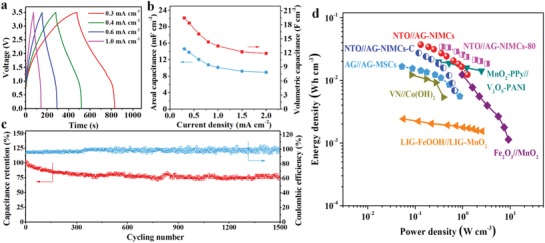
High‐temperature electrochemical performance of NTO//AG‐NIMCs at 80 °C. a) GCD profiles tested at current densities from 0.3 to 1 mA cm^−2^. b) Areal and volumetric capacitance versus current density. c) Cycling stability. d) Ragone plot of NTO//AG‐NIMCs, NTO//AG‐NIMCs‐80, NTO//AG‐NIMCs‐C, and AG//AG‐MSCs compared with previously reported hybrid MSCs.

The Ragone plot of NTO//AG‐NIMCs is exhibited in Figure 5d. It was calculated that NTO//AG‐NIMCs delivered a high volumetric energy density of 37.1 mWh cm^−3^, which was much superior to those of NTO//AG‐NIMCs‐C (27.3 mWh cm^−3^) and AG//AG‐MSCs (16.6 mWh cm^−3^). This value surpasses those of most reported hybrid MSCs (Table S1, Supporting Information), such as MnO_2_–polypyrrole//V_2_O_5_–polyaniline (19.8 mWh cm^−3^),^42^ Fe_2_O_3_//MnO_2_ (12 mWh cm^−3^),^43^ VN//Co(OH)_2_ (12.4 mWh cm^−3^),^44^ and laser‐induced graphene@FeOOH//laser‐induced graphene@MnO_2_ (2.4 mWh cm^−3^).^45^ Furthermore, NTO//AG‐NIMCs showed a higher power density of 1.2 W cm^−3^ than NTO//AG‐NIMCs‐C (0.7 W cm^−3^), further indicative of the favorable multidirectional ion diffusion. In addition, NTO//AG‐NIMCs displayed a high areal energy density of 27.8 µWh cm^−2^ and an areal power density of 0.9 mW cm^−2^ (Figure S18, Supporting Information). Areal energy density of NTO//AG‐NIMCs was much higher than those previously reported values (Table S2, Supporting Information), for instance, Ti_3_C_2_//Co–Al–layer double hydroxide (LDH) asymmetric micro‐supercapacitors (AMSCs, 10.8 µWh cm^−2^),^46^ polypyrrole (PPy)–multi‐walled carbon nanotube (MWCNT)//MnO_2_ AMSCs (12 µWh cm^−2^),^47^ and VN//MnO_2_ AMSCs (9 µWh cm^−2^).^48^

In summary, we have constructed all‐solid‐state planar NTO//AG‐NIMCs with the unique merit of multidirectional fast ion diffusion pathways, showing high volumetric performance, remarkable mechanical flexibility, and high‐temperature stability due to the use of nonflammable high‐voltage ionogel electrolyte and the advance of in‐plane separator‐free geometry. The exposed two side and top surfaces of the interdigital microelectrodes enable electrolyte ions to rapidly transfer from these interfaces, contributing to greatly enhanced power density of NTO//AG‐NIMCs. As a result, NTO//AG‐NIMCs exhibited a high volumetric energy density of 37.1 mWh cm^−3^ and robust high‐temperature electrochemical stability. Through further optimization of high‐capacitance electrode materials and establishment of abundant microchannels in microelectrodes with uniquely multidirectional ion transfer pathways, it is believed that microscale energy storage devices with interdigital in‐plane geometry will further offer notable energy density and power density simultaneously for some particular applications in smart and miniaturized electronics.

## Experimental Section

*Materials' Preparation*: Urchin‐like NTO was synthesized from the alkalization and oxidation of Ti_3_C_2_ through the hydrothermal process.^39^ Briefly, 2 g of Ti_3_AlC_2_ was selectively etched by 20 mL of HF (30%) with continuous stirring at room temperature for 24 h. After fully washed with water, Ti_3_C_2_ was obtained and dried under vacuum condition. To prepare NTO, 100 mg of Ti_3_C_2_ was mixed with 1 m NaOH (30 mL) and 0.68 mL of H_2_O_2_ (30 wt%) under stirring condition. Then the resultant dispersion was passed on to a Teflon‐lined stainless‐steel autoclave (50 mL). After hydrothermal treatment at 140 °C for 12 h, the white particles were collected through vacuum filtration and rinsed for three times, and dried at 60 °C for 24 h. Finally, the white powder of NTO was achieved.

AG was prepared by KOH activation of rGO aerogel at 800 °C. Typically, GO solution (6 mg mL^−1^) was freeze‐dried to form GO aerogel. Then, GO aerogel was rapidly reduced by self‐propagating combustion via a lighter to prepare rGO aerogel.^49^ Subsequently, 250 mg of rGO aerogel was immersed into 2 m KOH (100 mL) for 24 h. The resultant dispersion was then filtrated by vacuum deposition, and the collected black slurry was dried in a vacuum oven at 45 °C for 48 h. Subsequently, the obtained black powder at a horizontal tube furnace was heated to 800 °C for 1 h with an argon flow of 50 mL min^−1^. The resulting sample was washed for several times and completely dried. Later, the black dried powder was again heated to 800 °C for 2 h in argon to obtain the AG. EG nanosheets were prepared by electrochemical exfoliation of graphite at KOH solution, as previously reported.^50^, ^51^

*Ionogel Electrolyte*: NaTFSI was dissolved in P_14_TFSI to form 0.75 m NaTFSI–P_14_TFSI under fast stirring for 12 h. 0.2 g of PVDF‐HFP was added to acetone (2 mL) under continuous stirring until a transparent solution was formed. After that, NaTFSI–P_14_TFSI was mixed with PVDF‐HFP dispersion under stirring for 1 h. Finally, NaTFSI–P_14_TFSI–PVDF‐HFP ionogel electrolyte was obtained.

*Fabrication of NTO//AG‐NIMCs*: The customized mask was made of stainless steel mold with interdigital patterns, in which each side has four fingers with the length of 14 mm, width of 1 mm, and interspace of 0.5 mm. Initially, high‐conducting EG layer was formed from the deposition of EG dispersion (3 mL, 0.1 mg mL^−1^) on a nylon membrane (0.22 µm) via the interdigital mask. Then, NTO/EG dispersion (1 mL, 10 wt% EG, 0.5 mg mL^−1^) and AG/EG dispersion (1 mL, 10 wt% EG, 0.25 mg mL^−1^) were subsequently filtrated through each side of the mask to build the anode and cathode fingers, respectively. Afterward, the dilute EG dispersion (2 mL, 0.02 mg mL^−1^) was deposited on the anode and cathode from two sides of the mask to form high electronic conductive network. After NaTFSI–P_14_TFSI–PVDF‐HFP ionogel electrolyte was carefully coated on the project area of the microdevice and solidified, all‐solid‐state NTO//AG‐NIMCs were achieved. For comparison, AG//AG‐MSCs were also fabricated using the same method, except that AG was used as anode instead of NTO. The thickness of AG was the same as the cathode of NTO//AG‐NIMCs. To demonstrate the multidirectional ion diffusion, NTO//AG‐NIMCs‐C with a thick tight EG cover layer on the anode and cathode was constructed, where concentrated EG dispersion (2 mL, 0.1 mg mL^−1^) was used rather than the dilute one (2 mL, 0.02 mg mL^−1^).

*Material Characterizations*: Material characterizations of NTO, AG, ionogel electrolyte, and the microelectrodes of NTO and AG were carried out using SEM (JSM‐7800F), TEM (JEM‐2100), X‐ray diffraction (XRD X'pert Pro), nitrogen adsorption and desorption isotherm (Quadrasorb SI), four‐point probe equipment (RTS‐9), and thermogravimetric analysis (TGA, STA 449 F3). The ionic conductivity (σ) of ionogel electrolyte at different temperature was evaluated by the equation: σ = *L*/(*R* × *S*), where *L* (cm) and *S* (cm) are the length and geometric area of ionogel electrolyte film, respectively, *R* (Ω) is the ESR of EIS tested under a stainless steel/solid‐state ionogel electrolyte/stainless steel cell structure.

*Electrochemical Measurement*: The CV curves from 5 to 20 mV s^−1^, EIS spectra from 100 kHz to 0.01 Hz with an AC amplitude of 5 mV, and LSV curves at 2 mV s^−1^ under a cell architecture of stainless steel/ionogel electrolyte/sodium plate were recorded by electrochemical workstation (CHI760E). The GCD measurements at different current densities from 0.1 to 2 mA cm^−2^ and cycling stability were carried out by a LAND CT2001A battery tester. For high‐temperature measurement, NTO//AG‐NIMCs were kept at the tested temperature for 1 h until equilibration.

## Conflict of Interest

The authors declare no conflict of interest.

## Supporting information

Supplementary
