# Supplementary material for: All‐Solid‐State Planar Sodium‐Ion Microcapacitors with Multidirectional Fast Ion Diffusion Pathways
Source: Adv Sci (Weinh). 2019 Oct 4;6(23):1902147. doi: 10.1002/advs.201902147 (PMC6891900; doi:10.1002/advs.201902147)
Supplement: Supplementary file 1 — Supplementary [file ADVS-6-1902147-s001.pdf]

Copyright WILEY-VCH Verlag GmbH & Co. KGaA, 69469 Weinheim, Germany, 2019.

Supporting Information

for *Adv. Sci.,* DOI: 10.1002/advs.201902147

All-Solid-State Planar Sodium-Ion Microcapacitors with Multidirectional Fast Ion Diffusion Pathways

Shuanghao Zheng, Sen Wang, Yanfeng Dong, Feng Zhou, Jieqiong Qin, Xiao Wang, Feng Su, Chenglin Sun, Zhong-Shuai Wu, Hui-Ming Cheng, and Xinhe Bao*

Copyright WILEY-VCH Verlag GmbH & Co. KGaA, 69469 Weinheim, Germany, 2019.

Supporting Information

All-Solid-State Planar Sodium Ion Micro-Capacitors with Multi-Directional Fast Ion Diffusion Pathways

Shuanghao Zheng, Sen Wang, Yanfeng Dong, Feng Zhou, Jieqiong Qin, Xiao Wang, Feng Su, Chenglin Sun, Zhong-Shuai Wu, Hui-Ming Cheng, and Xinhe Bao*

Mr. S. Zheng, Ms. S. Wang, Dr. Y. Dong, Dr. F. Zhou, Ms. J. Qin, Ms. X. Wang, Mr. F. Su, Prof. C. Sun, Prof. Z.-S. Wu, Prof. X. Bao
Dalian National Laboratory for Clean Energy
Dalian Institute of Chemical Physics, Chinese Academy of Sciences
457 Zhongshan Road, Dalian 116023, China
E-mail: wuzs@dicp.ac.cn

Mr. S. Zheng, Prof. X. Bao
State Key Laboratory of Catalysis
Dalian Institute of Chemical Physics, Chinese Academy of Sciences
457 Zhongshan Road, Dalian 116023, China

Prof. H.-M. Cheng
Shenyang National Laboratory for Materials Science, Institute of Metal Research, Chinese Academy of Sciences, 72 Wenhua Road, Shenyang 110016, China

Prof. H.-M. Cheng
Shenzhen Geim Grpahene Center, Tsinghua-Berkeley Shenzhen Institute, 1001 Xueyuan Road, Shenzhen 518055, China

Mr. S. Zheng, Ms. S. Wang, Ms. J. Qin, Ms. X. Wang, Mr. F. Su
University of Chinese Academy of Sciences
19 A Yuquan Rd, Shijingshan District, Beijing 100049, China

Calculation

The specific areal capacitance and volumetric capacitance of NIMCs were evaluated through GCD profiles via the following equations (1) and (2):

$$\mathrm{C}_{area} = \frac{It}{\Delta U A_{device}} \quad (1)$$

$$\mathrm{C}_{volume} = \frac{It}{\Delta U V_{device}} \quad (2)$$

Where C_{area} (mF cm^{-2}) and C_{volume} (F cm^{-3}) are the calculated areal capacitance and volumetric capacitance, respectively. I (mA) is the discharge current. t (s) is the discharge time. ΔU (V) is the potential difference. A_{device} (cm^{-2}) and V_{device} (cm^{-3}) are the area and volume of two electrodes, respectively.

The volumetric energy density and power density of NIMCs were calculated by the formula (2) and (3):

$$\mathrm{E}_{volume} = \frac{I\int Udt}{V_{device}} \quad (3)$$

$$\mathrm{P}_{volume} = \frac{E_{volume}}{t} \times 3600 \quad (4)$$

Where U is potential (V), E_{volume} is volumetric energy density (mWh cm^{-3}), P_{volume} is volumetric power density (mW cm^{-3}).

The areal energy density and power density of NIMCs were evaluated by the equations (5) and (6):

$$\mathrm{E}_{area} = \frac{I\int Udt}{V_{area}} \quad (5)$$

$$\mathrm{P}_{area} = \frac{E_{area}}{t} \times 3600 \quad (6)$$

Where U is potential (V), E_{area} is volumetric energy density (μWh cm^{-2}), P_{area} is volumetric power density (mW cm^{-2}).

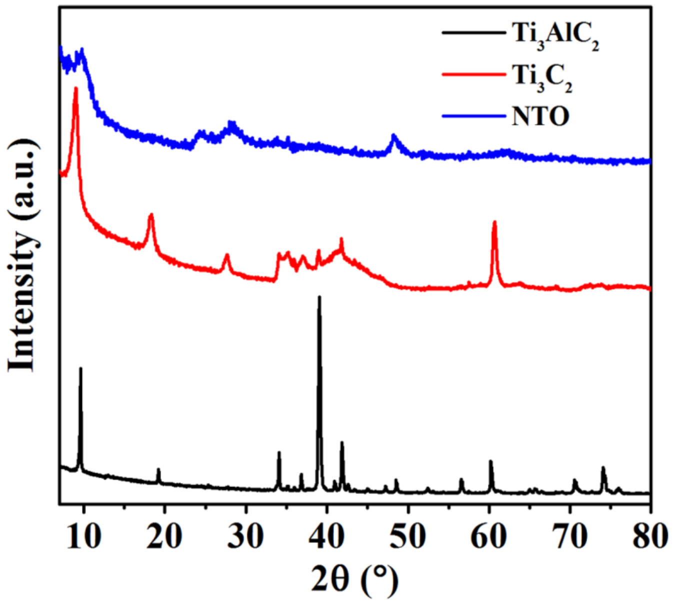

Figure S1. XRD patterns of Ti_3AlC_2, Ti_3C_2 and NTO.

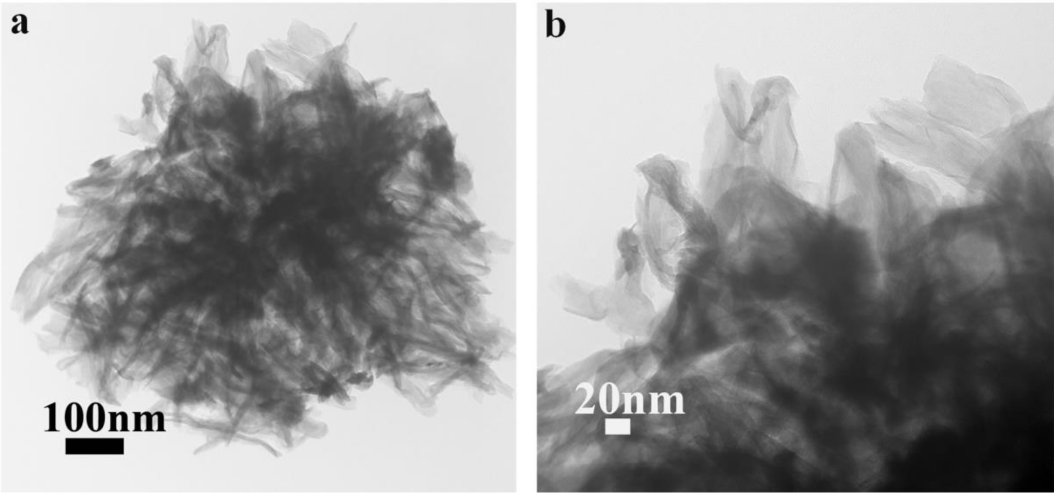

Figure S2. TEM images of NTO with low (a) and high (b) magnification.

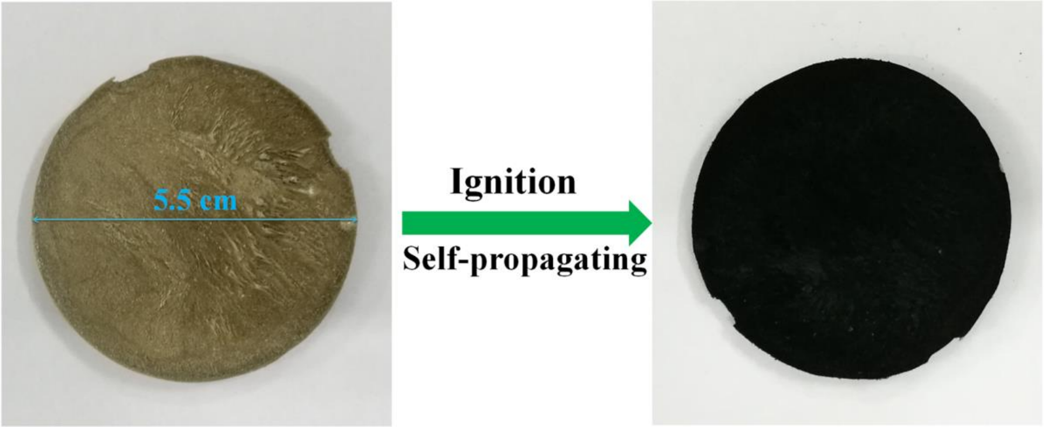

Figure S3. Photographs of brown GO aerogel (left) and black rGO aerogel (right), with a diameter of 5.5 cm. The color change from brown GO aerogel to black rGO aerogel indicates the efficient reduction by self-propagating combustion in seconds.

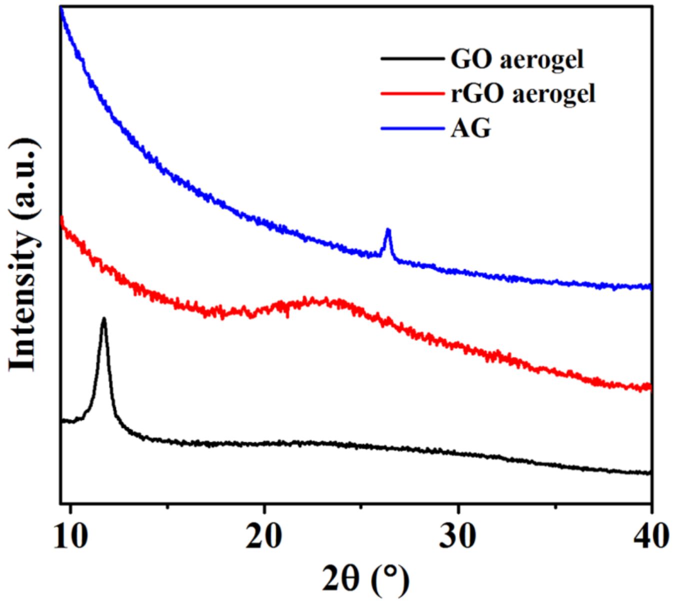

Figure S4. XRD patterns of GO aerogel, rGO aerogel and AG. The changed peaks indicated the structural evolution.

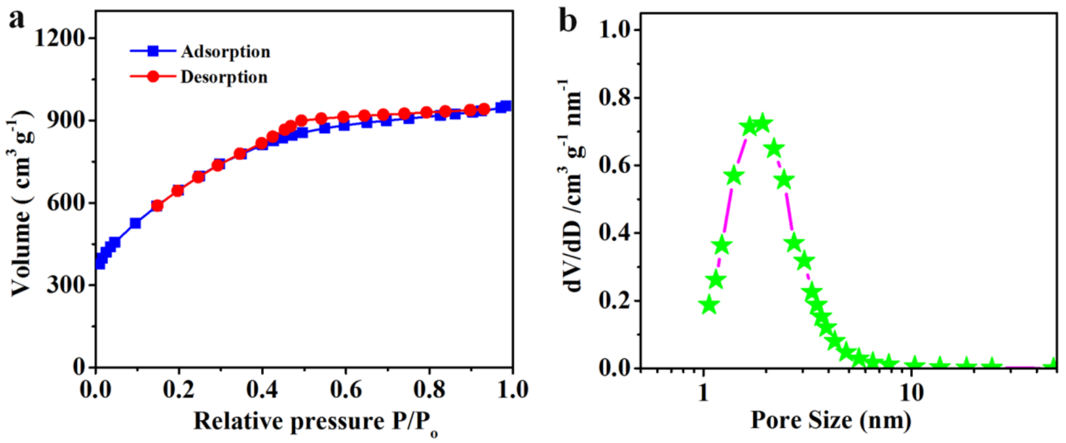

Figure S5. a) Nitrogen adsorption and desorption isotherm and b) pore size distribution of AG.

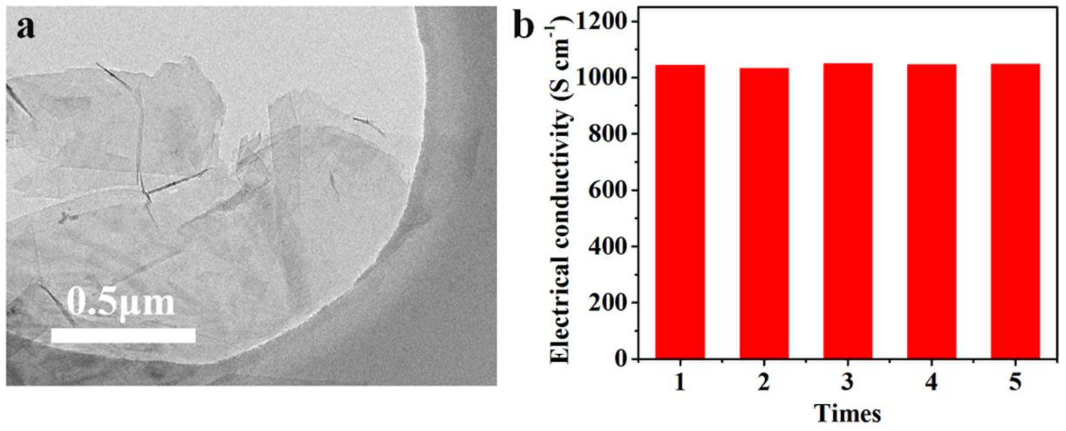

Figure S6. a) TEM image of EG nanosheets, showing transparent ultrathin layer of EG. b) Electronic conductivity of EG thin film.

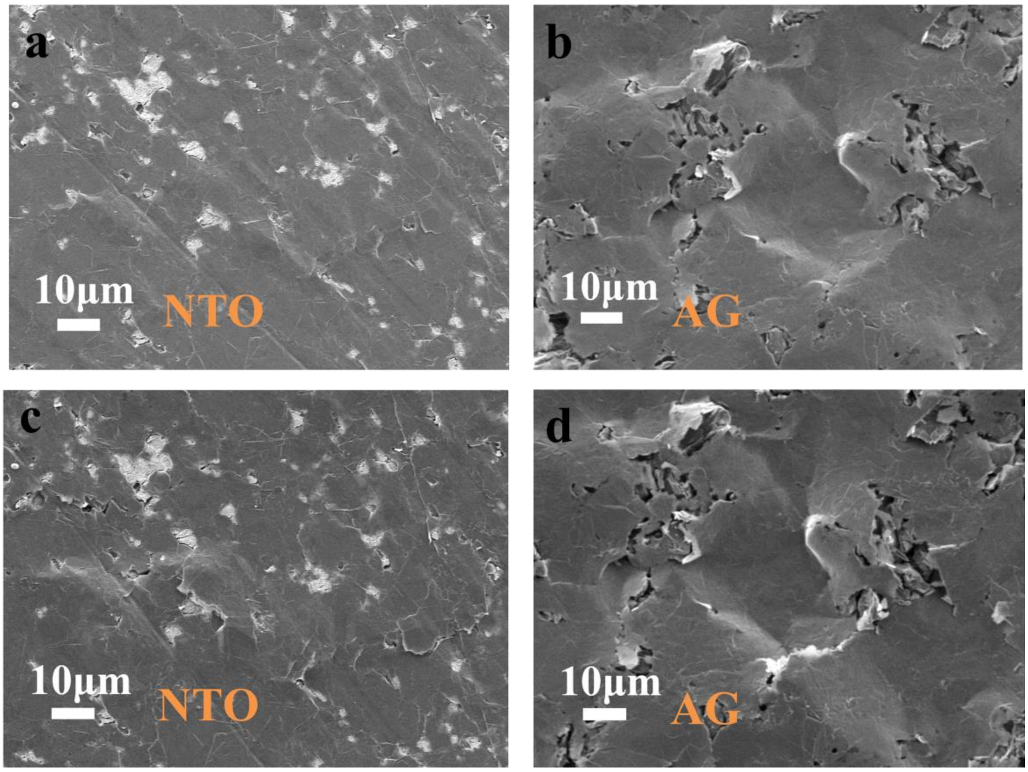

Figure S7. Top-view SEM images of NTO and AG microelectrode films (a,b) before and (c,d) after bending as a circle.

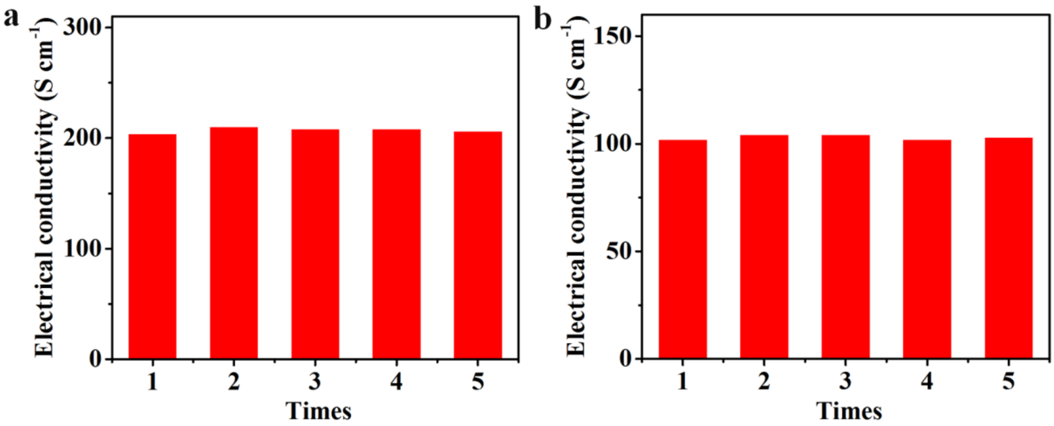

Figure S8. Electronic conductivity of a) anode and b) cathode tested for 5 times.

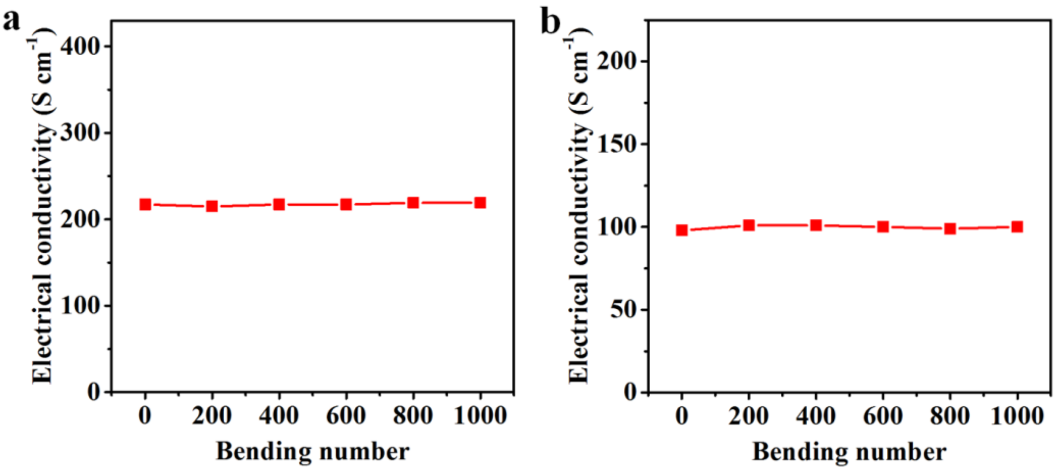

Figure S9. Electrical conductivities of NTO (a) and AG (b) microelectrodes as a function as bending number.

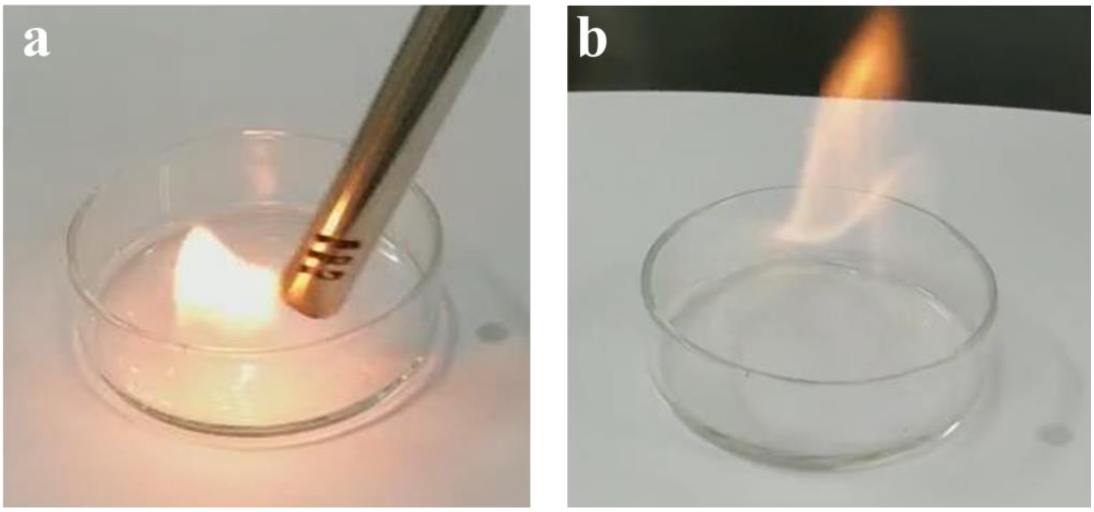

Figure S10. (a,b) Flammable test of commercial sodium ion gel electrolyte of $NaPF_6$ in diglyme mixed with PVDF-HFP.

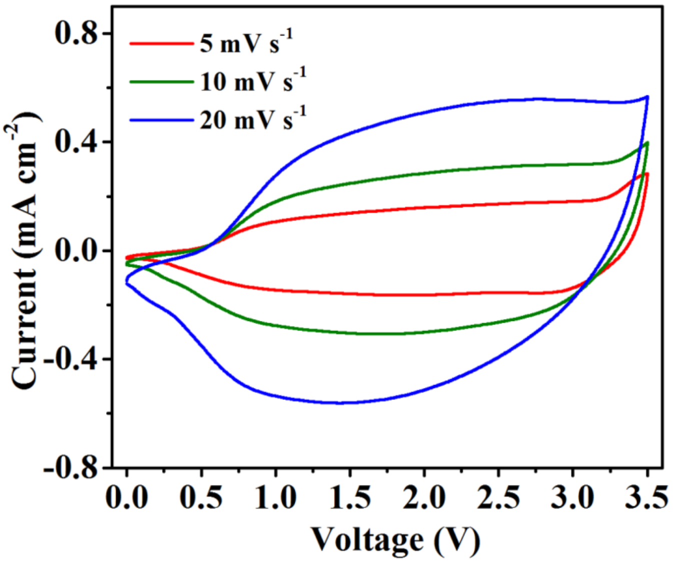

Figure S11. CV curves of NTO//AG-NIMCs tested at scan rates from 5 to 20 mV s^{-1}.

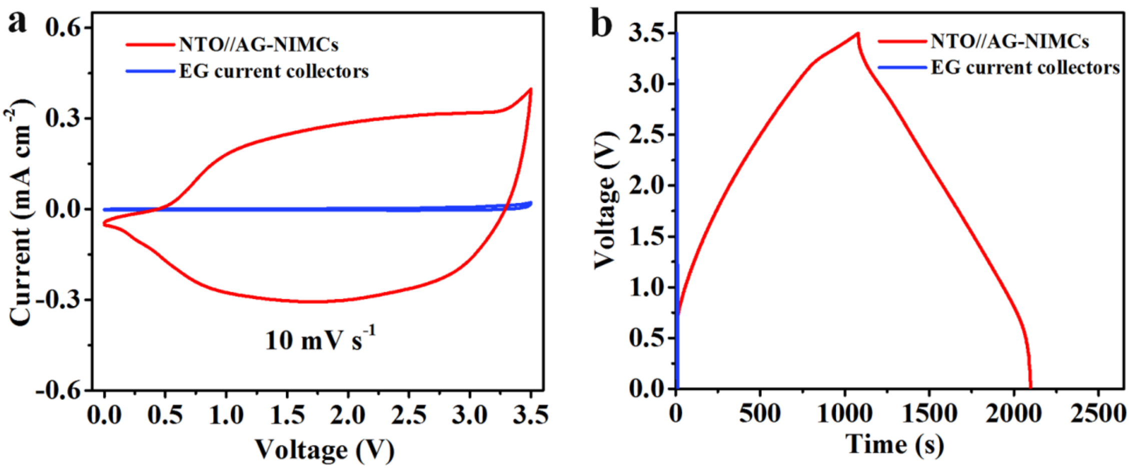

Figure S12. a) CV curves tested at 10 mV s^{-1} and b) GCD profiles obtained at 0.1 mA cm^{-2} of NTO//AG-NIMCs and EG current collectors. These results suggest that contribution of EG to capacitance can be negligible.

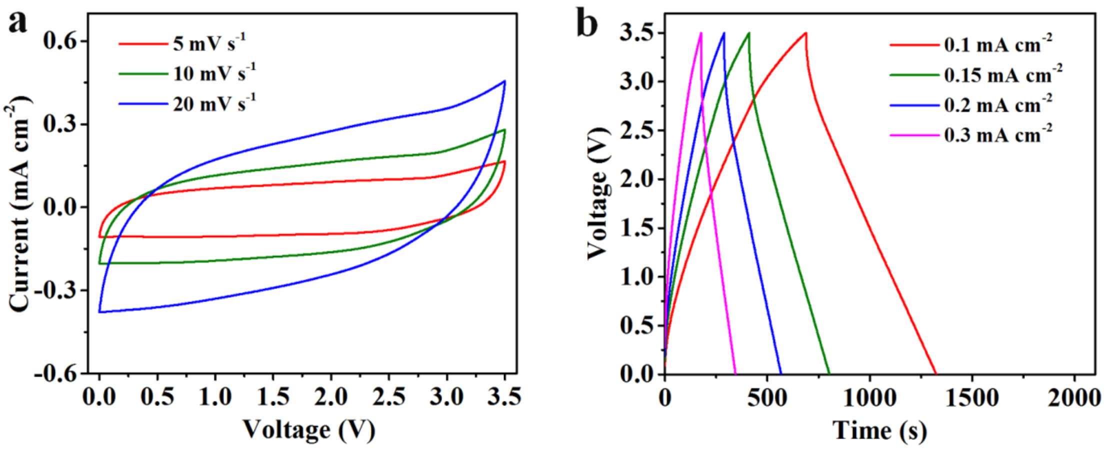

Figure S13. a) CV curves recorded from 5 to 20 mV s^{-1} and b) GCD profiles tested from 0.1 to 0.3 mA cm^{-2} of AG//AG-MSCs.

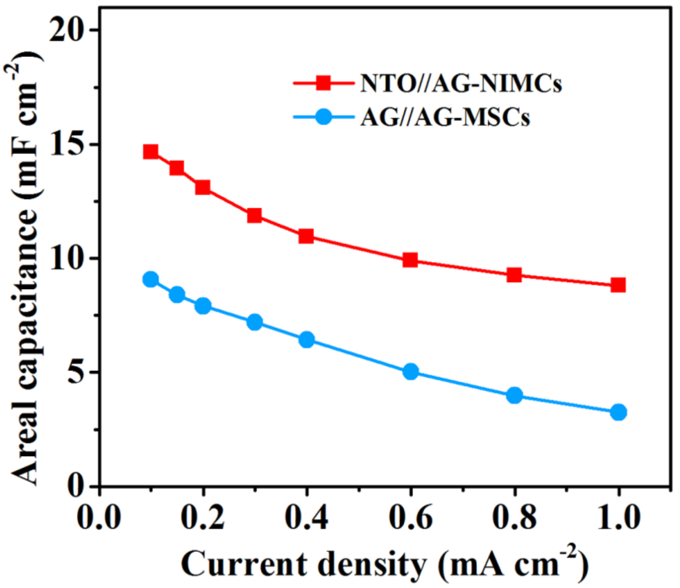

Figure S14. Areal capacitance of NTO//AG-NIMCs and AG//AG-MSCs as a function of current density.

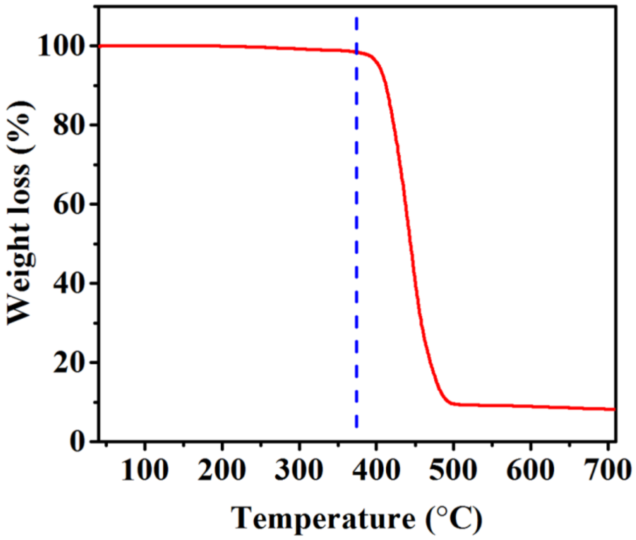

Figure S15. TG curve of ionogel electrolyte, showing that the ionogel electrolyte is thermally stable at the temperature of up to 370 °C.

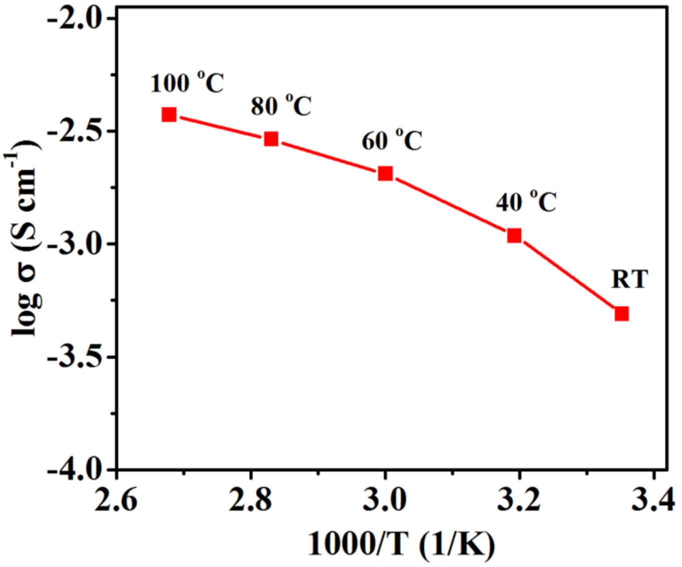

Figure S16. Ionic conductivity of ionogel electrolyte as a function of temperature.

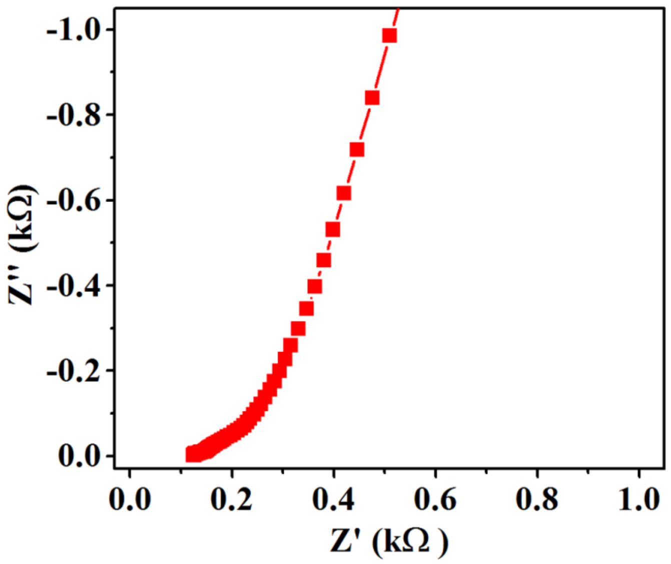

Figure S17. EIS spectrum of NTO//AG-NIMCs-80.

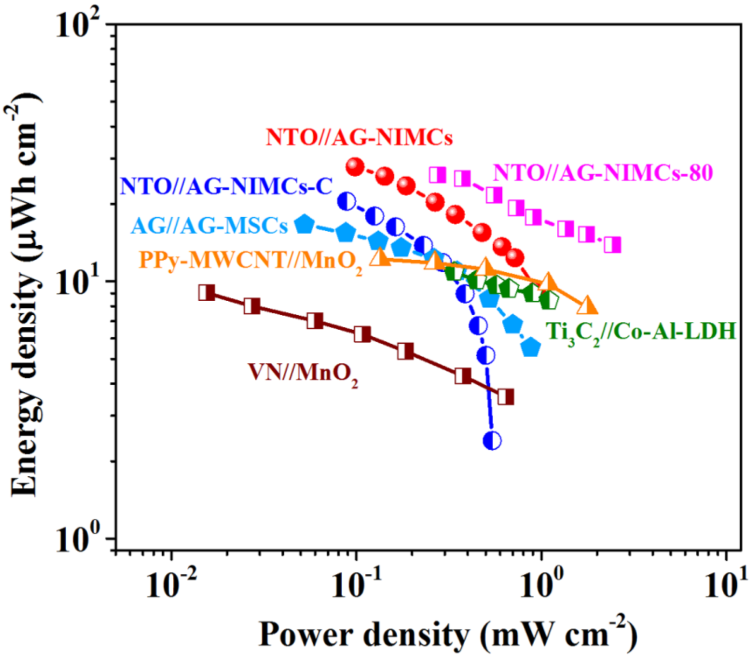

Figure S18. Ragone plots showing areal energy density and power density of NIMCs.

Table S1. Volumetric electrochemical performance comparison of NTO//AG-NIMCs with reported hybrid MSCs

Electrodes	Electrolyte	V	Cv (F cm^{-3})	Pv (mW cm^{-3})	Ev (mWh cm^{-3})	Refs
**NTO//AG**	NaTFSI P_{14}TFSI PVDF-HFP	0-3.5	19.5	1200	37.1	This work
**MnO_2-PPy// V_2O_5-PANI**	LiCl/PVA	0-1.6	/	2570	19.8	[1]
**Ti_3C_2//Co-Al-LDH**	6 M KOH	0.4-1.45	/	/	/	[2]
**LIG-FeOOH// LIG-MnO_2**	LiCl/PVA	0-1.8	5.4	2890	2.4	[3]
**EG//MP**	LiCl/PVA	0-1.8	14.6	1460	6.6	[4]
**VN//$Co(OH)_2$**	KOH/PVA	0-1.5	39.7	1750	12.4	[5]
**Fe_2O_3//MnO_2**	KOH solution	0-1.2	60	14800	12	[6]
**VN//MnO_2**	LiTFSI/SiO_2	0-2	38.8	1539	21.6	[7]
**RGO//GO/PANI**	H_3PO_4/PVA	0-1.2	25	25000	4.8	[8]

V: Voltage; Cv: Volumetric capacitance; Ev: Volumetric energy density; Pv: Volumetric power density. PPy: Polypyrrole; PANI: Polyaniline; PVA: Polyvinyl alcohol; LDH: Layer double hydroxides; LIG: Laser-induced graphene; EG: electrochemically exfoliated graphene; MP: MnO_2 nanosheets/PH1000;

Table S2. Areal electrochemical performance comparison of NTO//AG-NIMCs with reported hybrid MSCs

Electrodes	Electrolyte	V	C_A (mF cm^{-2})	Pv (mW cm^{-2})	Ev (μWh cm^{-2})	Refs
**NTO//AG**	NaTFSI P_{14}TFSI PVDF-HFP	0-3.5	14	0.9	27.8	This work
**LIG-FeOOH// LIG-MnO_2**	LiCl/PVA	0-1.8	22	12	9.6	[3]
**Ti_3C_2//Co-Al-LDH**	6M KOH	0.4-1.45	40	1.2	10.8	[2]
**AC//MnO_2**	0.2M K_2SO_4	0-1.5	2.8	-	10	[9]
**FeHCF-graphene// CuHCF-graphene**	LiCl/PVA	0-1.8	20	-	9	[10]
**VN//$Co(OH)_2$**	KOH/PVA	0-1.5	21	0.9	6.5	[5]
**PPy-MWCNT//MnO_2**	LiCl/PVA	0-1.6	22	2	12	[11]
**VN//MnO_2**	LiTFSI/SiO_2	0-2	16.1	0.6	9	[7]

C_A: Areal capacitance; E_A: Areal energy density; P_A: Areal power density; FeHCF: Fe hexacyanoferrates; CuHCF: Cu hexacyanoferrates;

References

[1] Y. Yue, Z. Yang, N. Liu, W. Liu, H. Zhang, Y. Ma, C. Yang, J. Su, L. Li, F. Long, Z. Zou, Y. Gao, *ACS Nano* **2016**, *10*, 11249.

[2] S. Xu, Y. Dall'Agnese, G. Wei, C. Zhang, Y. Gogotsi, W. Han, *Nano Energy* **2018**, *50*, 479.

[3] L. Li, J. Zhang, Z. Peng, Y. Li, C. Gao, Y. Ji, R. Ye, N. D. Kim, Q. Zhong, Y. Yang, H. Fei, G. Ruan, J. M. Tour, *Adv. Mater.* **2016**, *28*, 838.

[4] X. Shi, Z.-S. Wu, J. Qin, S. Zheng, S. Wang, F. Zhou, C. Sun, X. Bao, *Adv. Mater.* **2017**, *29*, 1703034.

[5] S. Wang, Z.-S. Wu, F. Zhou, X. Shi, S. Zheng, J. Qin, H. Xiao, C. Sun, X. Bao, *npj 2D Mater. Appl.* **2018**, *2*, 7.

[6] Z. Liu, X. Tian, X. Xu, L. He, M. Yan, C. Han, Y. Li, W. Yang, L. Mai, *Nano Res.* **2017**, *10*, 2471.

[7] J. Qin, S. Wang, F. Zhou, P. Das, S. Zheng, C. Sun, X. Bao, Z.-S. Wu, *Energy Storage Mater.* **2019**, *18*, 397.

[8] Y. Liu, B. Zhang, Q. Xu, Y. Hou, S. Seyedin, S. Qin, G. G. Wallace, S. Beirne, J. M. Razal, J. Chen, *Adv. Funct. Mater.* **2018**, *28*, 1706592.

[9] C. Shen, X. Wang, S. Li, J. g. Wang, W. Zhang, F. Kang, *J. Power Sources* **2013**, *234*, 302.

[10] Y. He, P. Zhang, M. Wang, F. Wang, D. Tan, Y. Li, X. Zhuang, F. Zhang, X. Feng, *Mater. Horiz.* **2019**, *6*, 1041.

[11] J. Gao, C. Shao, S. Shao, F. Wan, C. Gao, Y. Zhao, L. Jiang, L. Qu, *Small* **2018**, *14*, 1801809.
